# Assessment of the potential impact of resolving drug-related problems by clinical pharmacists in Japan: a retrospective observational study

**DOI:** 10.1186/s40780-021-00232-9

**Published:** 2021-12-06

**Authors:** Toshiya Oki, Sachi Ishii, Koya Furukawa, Aiko Shono, Manabu Akazawa

**Affiliations:** 1 Department of Public Health and Epidemiology, Meiji Pharmaceutical University, 2-522-1 Noshio, Kiyose, Tokyo, 204-8588 Japan; 2 Department of Pharmacy, IMS Miyoshi General Hospital, 974-3 Fujikubo, Miyoshi-machi, Iruma-gun, Saitama, 354-0041 Japan; 3 Division of Social Pharmacy and Regulatory Science, Showa Pharmaceutical University, 3-3165 Higashitamagawagakuen, Machida, Tokyo, 194-8543 Japan

**Keywords:** Drug-related problem, Pharmacist intervention, Potential impact, Medication review, Clinical pharmacist, Japan

## Abstract

**Background:**

Clinical pharmacists play a role in limiting the disadvantages of pharmacotherapy for patients by detecting and resolving drug-related problems (DRPs) through medication reviews. Although their contributions to patient care have been analyzed and understood in various countries, the role of Japanese clinical pharmacists in this context remains to be clearly elucidated. Thus, in this study, we aimed to elucidate the detection of DRPs by clinical pharmacists and determine the potential impact of pharmacist interventions in Japan.

**Methods:**

This study was conducted in a 273-bed hospital and targeted hospitalized patients over a period of 6 months. DRPs detected by clinical pharmacists during the study period were investigated and classified into 10 types. Furthermore, medications were categorized according to the Anatomical Therapeutic Chemical classification. A review committee consisting of two pharmacists independently reviewed the pharmacist interventions on a six-point scale (extremely significant, very significant, significant, somewhat significant, no significance, adverse significance) according to the potential impact on patient care.

**Results:**

During the study period, 1711 patients (mean age: 71.2 years, 54.1% male) were included, and 2149 DRPs were detected (1.26 DRPs/patient). Pharmacists intervened in all the DRPs detected. The most common DRP was supratherapeutic dosage (19.3%), followed by untreated indication (18.1%). The most common medication classification causing DRPs was “Antiinfectives for Systemic Use” (25.1%), followed by “Alimentary Tract and Metabolism” (19.9%). Most of the pharmacist interventions (99.6%) were rated “somewhat significant” or more significant, of which 1.1% were rated “extremely significant,” and none were rated as “adverse significance.”

**Conclusions:**

Our results show that in Japan, as in other countries, clinical pharmacists detect and resolve DRPs in hospitalized patients through medication review. Our findings also show that clinical pharmacists have a positive impact on patient care and suggest the need for their involvement.

## Background

Drug-related problems (DRPs) are events or circumstances involving drug therapy that potentially or actually interfere with desired health outcomes [1]. DRPs are related to causes including the selection of the drug, drug form, and dosage schedule; treatment duration; logistics of the prescribing and dispensing process; way of drug administration; and patient behavior [1]. Over the past few decades, the number of drugs available on the market has increased markedly [2]. While this has expanded the scope of drug treatments it has also caused significant challenges in managing drug therapy. This may also be a possible cause of DRPs.

Medication review is a means of detecting and resolving DRPs and it is defined as “a structured evaluation of a patient’s medicines with the aim of optimizing medicines use and improving health outcomes. This entails detecting DRPs and recommending interventions” [3]. Medication review may be implemented as a self-review by the prescriber; however, it is generally implemented as an independent review by the pharmacist [4]. In hospitals, clinical pharmacists can make direct changes in treatment through interventions as part of a medication review. In other words, clinical pharmacists play a role in limiting the disadvantages of pharmacotherapy for patients.

Studies assessing the impact of clinical pharmacists in hospital settings have shown the following results. Pharmacists’ participation in medical rounding teams in general wards has contributed to a significant reduction in preventable adverse drug events [5]. Clinical pharmacy services and pharmacy staffing are associated with reduced mortality rates [6]. Pharmacist intervention may have a positive effect on the length of hospital stay, number of adverse drug events, and drug-related readmissions [7–9]. Moreover, pharmacist interventions have been proven to save costs [7, 10]. One method to characterize the value of a pharmacist’s activities is to rank the pharmacist intervention on a six-point scale according to the potential impact on patient care [11]. Studies assessing pharmacist intervention using this or similar scales have reported positive results attributable to the activities of clinical pharmacists [12–19].

The relevance of clinical pharmacists has increased in Japan. In March 2010, the Ministry of Health, Labour and Welfare issued the “Report for Enhancing the Team Approach to Provide Healthcare,” which stated the roles and functions that pharmacists should undertake per the current laws and regulations [20]. This led to clinical pharmacists performing medication reviews as part of their role. The documentation of pharmacist intervention and assessment of the potential impact of this intervention are necessary for the further development and retention of clinical pharmacists. Although the potential impact of pharmacist intervention has been reported in other countries, it has not been reported in Japan [11, 15, 17, 21]. In addition, previous studies have analyzed DRPs using their original medication classifications [22, 23], and a few studies have analyzed DRPs using the Anatomical Therapeutic Chemical (ATC) classification, which is commonly used worldwide [13, 14]. However, only a few studies have focused on the relationship between DRP and ATC classification [19]. Clarifying the relationship between DRP and ATC classification will be very useful for ensuring safe and effective pharmacotherapy. Therefore, in this study, we aimed to elucidate the detection of DRPs by medication review and determine the potential impact of pharmacist intervention.

## Methods

### Setting

IMS Miyoshi General Hospital is a 273-bed secondary emergency hospital with 27 clinical departments, allocated between ten internal medical and 17 surgical departments, which is located in a suburb of Tokyo, Japan. In 2009, the hospital began to assign clinical pharmacists specialized in patient care to some wards as dedicated staff, and in 2010, they were assigned to all wards. Clinical pharmacists participate in medical rounds as needed and conduct rounds on their own. They review medications immediately upon prescription. On nights and weekends when dedicated clinical pharmacists are unavailable, prescriptions are reviewed by other pharmacists and re-reviewed by the dedicated clinical pharmacists the following day. Clinical pharmacists record DRPs detected through these activities, pharmacist interventions for DRPs, and physicians’ acceptance of the interventions in the pharmacist records.

### Design

This retrospective observational study was conducted to elucidate the detection of DRPs via medication review by clinical pharmacists and determine the potential impact of pharmacist interventions. Patients who were discharged from the hospital between January 2018 and June 2018 were included in the study. Patients for whom a clinical pathway was applied were excluded because the associated conditions, such as a predetermined length of stay and medical treatment, differed significantly from those of other hospitalized patients.

### Data collection

The database containing patient and pharmacist intervention data was constructed by reviewing electronic medical and pharmacists’ records. Patient data included sex, age, clinical department, number of medications at admission, and length of stay. Pharmacist intervention data included medication, ATC classification, DRP classification [24] (Table 1), information provided to the physician through pharmacist intervention, pharmacist intervention classification [24] (Table 1), and physicians’ acceptance. Pharmacist intervention was judged as “accepted” only if it was immediately applied by the physician. Partially accepted pharmacist interventions (e.g., a dose reduction applied by a discontinuation recommendation and a dose increase to a different dosage than the recommended amount) were judged as “not accepted.” Only the first level of the ATC classification was used in this study. Medications with multiple ATC classifications were assigned to one classification based on the prescribing intent. Medications without ATC classification were assigned to the ATC classification of a similar medicine. Chinese medicines were not assigned an ATC classification because they do not have an ATC classification.

**Table 1:** Classifications of DRPs and pharmacist interventions and potential impacts

DRP classification	
1. Non conformity to guidelines or contraindication	
2. Untreated indication	
3. Subtherapeutic dosage	
4. Supratherapeutic dosage	
5. Drug without indication	
6. Drug interaction	
7. Adverse drug reaction	
8. Improper administration	
9. Failure to receive drug	
10. Drug monitoring	
Pharmacist intervention classification	
1. Addition of a new drug	
2. Drug discontinuation	
3. Drug switch	
4. Change of administration route	
5. Drug monitoring	
6. Administration mode optimization	
7. Dose adjustment	
Potential impact classification	
1. Extremely significant-information qualified by life and death situation.	
2. Very significant-recommendation qualified by a potential or existing major organ dysfunction.	
3. Significant-recommendation would bring care to a more acceptable and appropriate level (i.e., standard of practice).	
4. Somewhat significant-benefit of the recommendation to the patient could be neutral depending on professional interpretation (to be differentiated from rank 3 where a standard of practice would support the recommendation).	
5. No significance-recommendation is informational (not specifically related or meaningful to the patient in question.)	
6. Adverse significance-recommendation supplied by the clinician may lead to adverse outcome.	

*DRPs* Drug-related problems

### Assessment of the potential impact of resolving DRPs

A review committee comprising two pharmacists was formed to assess the potential impact of pharmacist interventions. The committee independently reviewed the data recorded in the database and assessed the potential impact of each pharmacist intervention (Table 1) [11]. It did not have access to data on physicians’ acceptance of the interventions because it could affect the assessment. If there was a discrepancy between the committee members’ assessments, they discussed between themselves and decided on one rating.

### Statistical analyses

Continuous data on age are shown as the mean ± standard deviation values, whereas those such as the number of medications at admission and length of stay are shown as median values and interquartile range. Categorical data are presented as numbers (percentages). All analyses were performed using Microsoft Excel 2016 software.

## Results

A total of 1711 patients were included in the study (Table 2). The majority of the participants were elderly (mean age of 71.2 ± 16.5 years), and there were slightly more males (54.1%) than females. Overall, 2149 DRPs were detected (1.26 DRP/patient), and pharmacists intervened in all of the detected DRPs.

**Table 2:** Characteristics of the study population (*n* = 1711)

Sex (male/female)	
Male, n (%)	925 (54.1)
Female, n (%)	786 (45.9)
Age, years	71.2 ± 16.5
Type of clinical department	
Internal medicine, n (%)	653 (38.2)
Surgery, n (%)	1058 (61.8)
Number of medications at admission, n	4 (2–8)
Length of stay, days	12 (6–26)

Continuous data on age are shown as the mean ± standard deviation values, whereas continuous data such as the number of medications at admission and length of stay are shown as median values and interquartile range. Categorical data are shown as numbers (percentages)

### Detection of DRPs

**Fig. 1:**
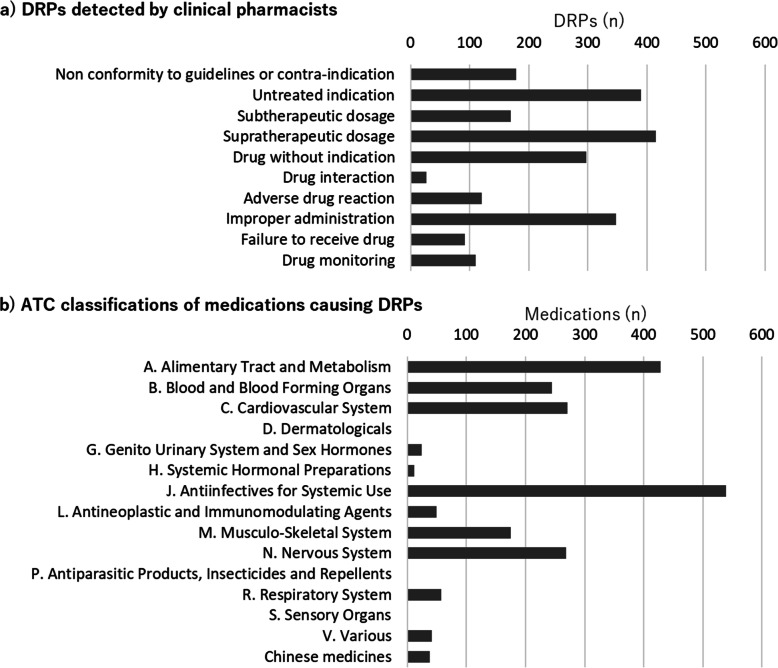
**a**) DRPs detected by clinical pharmacists, **b**) ATC classifications of medications causing DRPs (*n* = 2149). DRPs: Drug-related problems; ATC: Anatomical Therapeutic Chemical

The DRPs detected by the clinical pharmacists are shown in Fig. 1a. The most common DRPs were supratherapeutic dosage (*n* = 415, 19.3%), untreated indication (*n* = 390, 18.1%), improper administration (*n* = 348, 16.2%), and drug without indication (*n* = 298, 13.9%). Drug interactions were infrequently detected (*n* = 27, 1.3%). The ATC classifications of the medications that caused DRPs are shown in Fig. 1b. The most common ATC classifications were “Antiinfectives for Systemic Use” (*n* = 539, 25.1%) and “Alimentary Tract and Metabolism” (*n* = 428, 19.9%). “Systemic Hormonal Preparations” (*n* = 12, 0.6%), “Genito Urinary System and Sex Hormones” (*n* = 24, 1.1%), and “Chinese Medicine” (*n* = 38, 1.8%) were detected infrequently. “Dermatologicals,” “Antiparasitic Products, Insecticides and Repellents”, and “Sensory Organs” were not detected. The cross-tabulation of DRPs and ATC classifications is shown in Table 3.

**Table 3:** Relationship between DRPs and ATC classification

DRP	ATC classification											
	A	B	C	G	H	J	L	M	N	R	V	CMs
Non conformity to guidelines or contraindication	37	31	21	3	1	41	0	28	8	0	7	2
Untreated indication	117	66	76	10	3	23	1	25	43	11	7	8
Subtherapeutic dosage	18	31	9	1	1	66	9	3	29	1	1	1
Supratherapeutic dosage	74	46	26	0	2	166	14	36	39	6	4	2
Drug without indication	63	19	71	5	1	11	0	55	31	23	8	11
Drug interaction	4	1	1	0	0	15	0	1	1	0	4	0
Adverse drug reaction	21	5	30	2	0	10	3	11	30	1	1	6
Improper administration	62	29	30	1	4	109	21	7	74	5	4	2
Failure to receive drug	32	3	7	2	0	2	2	9	14	10	5	6
Drug monitoring	0	14	0	0	0	96	0	0	0	0	0	0
ATC classification												
A. Alimentary Tract and Metabolism					L. Antineoplastic and Immunomodulating Agents							
B. Blood and Blood Forming Organs					M. Musculo-Skeletal System							
C. Cardiovascular System					N. Nervous System							
G. Genito Urinary System and Sex Hormones					R. Respiratory System							
H. Systemic Hormonal Preparations					V. Various							
J. Antiinfectives for Systemic Use					CMs: Chinese Medicines							

*DRPs* Drug-related problems, *ATC* Anatomical Therapeutic Chemical

### Pharmacist interventions and physicians’ acceptance

**Fig. 2:**
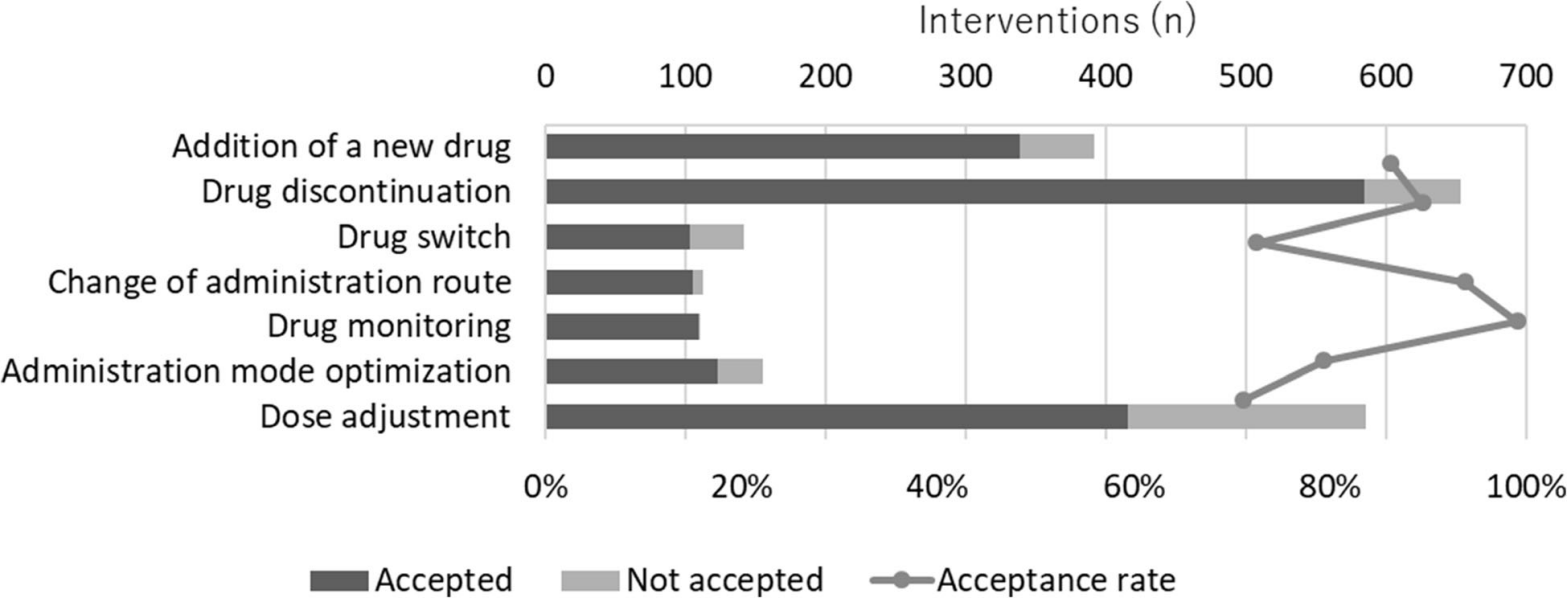
Pharmacist interventions and physicians’ acceptance of these interventions (*n* = 2149)

Pharmacist interventions and physician acceptance of the interventions are shown in Fig. 2. Of the 2149 pharmacist interventions, 1778 were accepted (acceptance rate = 82.7%). The most common pharmacist interventions performed to resolve DRPs were drug discontinuation (*n* = 653, 30.4%), dose adjustment (*n* = 585, 27.2%), and addition of a new drug (*n* = 392, 18.2%). In terms of the acceptance rate, the most frequently accepted interventions were drug monitoring (99.1%), change in the administration route (93.8%), and drug discontinuation (89.4%), while dose adjustment (71.1%) was infrequently accepted.

### Potential impact of pharmacist interventions

**Fig. 3:**
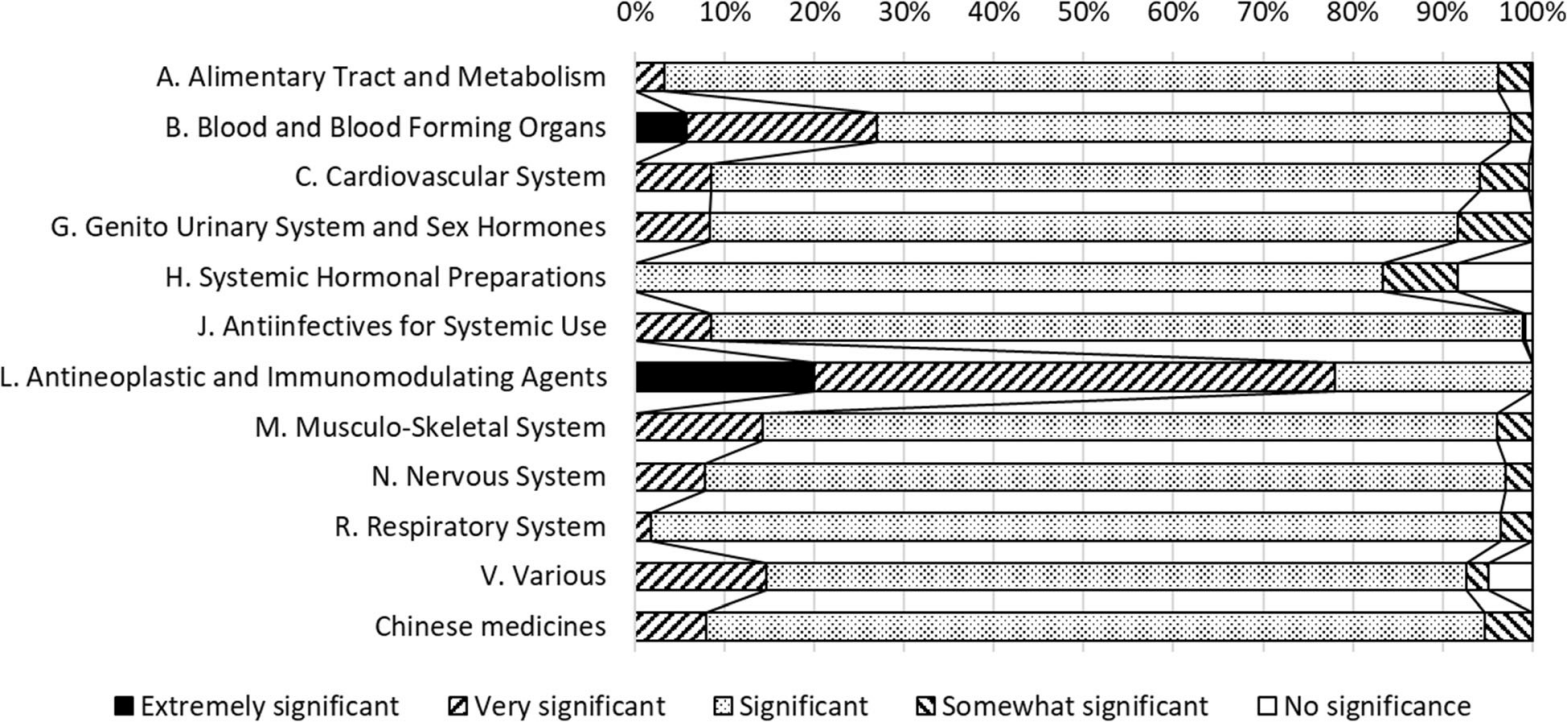
Relationship between the potential impact of pharmacist interventions and ATC classification (*n* = 2149)

Percentages of each assessment and examples of pharmacist interventions with each of these ratings are shown in Table 4. Most of the pharmacist interventions (98.9%) were rated between “very significant” and “no significance.” One percent of pharmacist interventions were rated “extremely significant.” None were rated “adverse significance.” The relationship between the potential impact of the interventions and ATC classifications is shown in Fig. 3. ATC classifications that were frequently rated higher than “very significant” were “Antineoplastic and Immunomodulating Agents” (78.0%) and “Blood and Blood Forming Organs” (26.9%). In contrast, the ATC classifications that were frequently rated lower than “somewhat significant” were “Systemic Hormonal Preparations” (16.7%) and “Genito Urinary System and Sex Hormones” (8.3%).

**Table 4:** Potential impact of 2149 pharmacist interventions assessed by the review committee

Potential impact	n (%)	Examples of pharmacist intervention
1. Extremely significant	24 (1.1)	A patient taking cilostazol for a history of stroke was admitted for surgery. Cilostazol was discontinued before the surgery but was not restarted since the risk of bleeding decreased after surgery. With pharmacist intervention, cilostazol was restarted. Restarting (adding) cilostazol reduces the risk of cerebral infarction recurrence.
2. Very significant	222 (10.3)	A patient with a history of penicillin allergy was prescribed penicillin to treat an infection. With pharmacist intervention, penicillin was switched to another medication before it was administered. Avoiding the administration of penicillin to a patient with a history of penicillin allergy inhibits the recurrence of allergy.
3. Significant	1833 (85.3)	A patient receiving treatment for infection had a vancomycin trough value of less than 10. With pharmacist intervention, the vancomycin dose was adjusted and a trough value of 15 was achieved. Appropriate vancomycin trough values can support the treatment of infection.
4. Somewhat significant	61 (2.8)	A patient receiving treatment for hypertension prior to hospitalization was not taking medication properly before admission. With pharmacist intervention, it was decided to discontinue the antihypertensive medication and monitor the patient’s condition.
5. No significance	9 (0.4)	Ceftriaxone was prescribed for intravenous infusion without dissolution. With pharmacist intervention, saline was added to ceftriaxone. Ceftriaxone should be dissolved before administration.
6. Adverse significance	0	None reported.

## Discussion

In Japan, the relevance of clinical pharmacists is increasing and their role needs to be expanded. This study was aimed at elucidating pharmacist interventions and assessing their potential impact on the further development and retention of clinical pharmacists. The results showed that clinical pharmacists detected an average of > 1 DRP per patient through pharmacist intervention, suggesting the need for clinical pharmacists. To our knowledge, this is the first study in Japan to analyze interventions by clinical pharmacists and assess their potential impact.

### Detection of DRPs

The most frequently detected DRP was supratherapeutic dosage. It was also the most frequently detected DRP in other studies using the same classification (24.0% [12] and 32.7% [13]). In another study of DRPs in Japan, overdosage was reported to be the most frequently detected DRP (24.6%), although it used different classifications [23]. In pharmacotherapy, it is known that the dosage needed decrease according to the physiological functions that decline with age. The mentioned studies focused on the clinical activities of pharmacists in hospitalized patients, and the DRP may be explained by the fact that the majority of the patients were elderly (mean age of the patients in this study: 71.2 years, mean age of the patients in the study by Yailian et al. [12]: 65 years; patients’ mean age in the studies by Loustalot et al. [13] and Tasaka et al. [23] were not shown). In Japan, 13.3 million people have chronic kidney disease, and the prevalence of the disease is associated with older age [25]. Differently from our study, a report involving adults (42.6 ± 18.3 years) was characterized by the fact that “dose too low” was the most frequently detected DRP [22]. These findings support the hypothesis that the drug dosage needed decreases according to the physiological functions that decline with age.

In contrast to other studies, this study was characterized by a high number of untreated indications. This may be due in part to the fact that clinical pharmacists accompanied physicians on their rounds and conducted rounds on their own, making it easier to detect this type of DRPs. Another contributing factor may be that surgeries are performed at the hospital and pharmacists are involved in the perioperative period to prevent patients from forgetting to restart medications that were discontinued before surgery. Considering the overall distribution of the DRPs, the results of different studies [12, 13] varied. This is thought to be due to the different scales and departments of the hospitals where the said studies were conducted as well as the different medications frequently used in different departments and diseases, resulting in the detection of different DRPs.

The relationship between DRPs and ATC classification is shown in Table 3. The most frequent ATC classification associated with DRPs was “Antiinfectives for Systemic Use,” which may be attributable to the fact that many dosing recommendations (including therapeutic drug monitoring) are based on renal function. This can be explained by the relationship between the dosage and aging. In studies that analyzed the frequency of DRP by ATC classification, DRPs associated with “Antiinfectives for Systemic Use” were the second [13] or fourth [14] most common. “Nervous System,” which was the most frequent DRP in these studies, was the fourth most frequent DRP in this study. Although the DRP classification and ATC classification were used in different ways, “inappropriate dose” and “inappropriate route or form of drug administration” were frequently detected in “Antiinfectives for Systemic Use” in a previous study that showed the relationship between them [19]. This result is similar to our study. The second most frequent ATC classification associated with DRPs was “Alimentary Tract and Metabolism.” Since these medications are commonly used, unnecessary or duplicate dosing is likely. In studies that analyzed the frequency of DRP by ATC classification, “Alimentary Tract and Metabolism” was the third [13] or fifth [14] most common. In our study, as in previous reports [13, 14, 19], “Dermatologicals”, “Antiparasitic Products, Insecticides and Repellents”, and “Sensory Organs” are features that were not detected at all, or rarely. However, there may be additional opportunities for pharmacist interventions.

These similarities were observed despite the fact that the study was conducted in different hospital settings and departments (a French teaching hospital with 714 beds [13], an emergency department with 70 beds at a 400 bed-hospital in Spain [14], acute care internal medicine wards in leading medical centers in Switzerland [19]). This may be explained by the frequency of prescription of the ATC concerned. A study focusing on surgery was characterized by a high frequency (73.8%) of DRPs associated with “Antibiotic / Anti-microbial” [22]. In our study with a large number of surgical patients (61.8%), DRPs associated with “Antiinfectives for Systemic Use” were also found with the highest frequency (25.1%). A study focusing on rheumatology was characterized by the highest frequency (26.3%) of DRPs associated with “Analgesics and Anti-inflammatory drugs” [12].

### Pharmacist interventions and physicians’ acceptance

Physician acceptance rates for pharmacist interventions are affected by a variety of factors, including the patient, medication, physician–pharmacist relationships, health care system, and roles required of pharmacists. In this study, the overall physician acceptance rate was 82.7%, which is similar to that in previous studies (57.6–90.0%) [12, 13, 15–19, 22, 26]. This study can be said to have no outstanding differences compared to those in these previous studies. The most accepted pharmacist intervention in this study was drug discontinuation. This may be because the issue of polypharmacy has been discussed in recent years [27] and because the study was conducted in a hospital that is concerned about polypharmacy. Hence, both physicians and pharmacists were in a situation where they agree on drug discontinuation [28]. One study showed that the odds ratio for adverse drug reactions was significantly higher in older adults taking six or more medications [29]. Discontinuation of medications in patients with polypharmacy may help avoid preventable adverse drug reactions. The intervention with the lowest acceptance rate was dose adjustment, which may be because the acceptance decision was made immediately and strictly. Dose adjustments that differed from pharmacist recommendations were judged as not accepted.

### Potential impact of pharmacist interventions

In this study, the potential impact of the pharmacists’ intervention was between “very significant” and “no significance” in over 95% of the cases. The results were generally similar to those of previous studies that assessed pharmacist intervention using the same classifications, although the composition was different [11, 15, 17, 21]. It can be assumed that pharmacist interventions and the assessment of potential impacts were implemented in situations comparable to those in which the previous studies were conducted. The high rates of “Antineoplastic and Immunomodulating Agents” and “Blood and Blood Forming Organs” observed in relation to potential impact and ATC classification may be due to the characteristics of the medications. Because both “Antineoplastic and Immunomodulating Agents” and “Blood and Blood Forming Organs” are associated with life-threatening conditions, these medications are defined as “high-risk drugs” in Japan and treated strictly, including in terms of prescription, administration, and follow-up care. “Systemic Hormonal Preparations” and “Genito Urinary System and Sex Hormones” were the drugs for which pharmacist interventions were more frequently rated as less significant compared to “somewhat significant”; however, these are ATC classifications for which DRPs were rarely detected. The frequency of the use of medications associated with these ATC classifications at IMS Miyoshi General Hospital is unknown; however, the frequency of use and pharmacists’ experience with those medications must be considered. The number of DRPs detected in this study did not allow for a full discussion in this context.

### Limitations

We assessed the potential impact of the rating scale used in previous studies. However, there is no clear definition for this assessment, which therefore, depends on the evaluator. Thus, this result alone may not necessarily indicate equivalence between our study and similar studies in other countries. Furthermore, although this study was conducted by a review committee consisting only of pharmacists, differences in the assessment by profession were noted. In particular, differences in assessments between physicians and pharmacists have been observed in previous studies, which may be explained by differences in the perception of iatrogenic risk for patients by professionals [12]. In addition, in the absence of pharmacist intervention, DRPs may be resolved by physicians themselves, and unresolved DRPs do not always have a negative outcome for patients. Therefore, it would be difficult to make a clear comparison between the presence and absence of pharmacist interventions.

This study was conducted in a single hospital and was dependent on its characteristics of the hospital. For example, because of the limited number of departments at the hospital (e.g., no pediatrics, obstetrics, or hematology department) and the large number of elderly patients, the results observed in this study do not reflect the DRPs in the Japanese healthcare setting as a whole. Depending on the department, DRPs associated with “Dermatologicals,” “Antiparasitic Products, Insecticides and Repellents,” and “Sensory Organs” that were not reported in this study may also be detected. However, this study may drive the documentation of the pharmacists’ contribution to pharmacotherapy in other institutions in the future. For further development and retention of clinical pharmacists, it is necessary to accumulate documentation of pharmacist interventions in multi-center settings and assess their potential impact.

## Conclusions

We showed that in Japan, as in other countries, clinical pharmacists detect and resolve DRPs in hospitalized patients through medication review. The assessment of the potential impact of pharmacist interventions characterized the activities of clinical pharmacists and suggested the need for the inclusion of clinical pharmacists to improve the quality of patient care. The results of this study provide useful knowledge for understanding DRPs and pharmacist interventions in Japan, where the population is aging. The findings of this study may help ensure safe and effective pharmacotherapy in Japan and other countries with aging populations.

## Data Availability

All data generated or analyzed during this study are included in this article.

## Abbreviations

DRPs: Drug-related problems
ATC: Anatomical Therapeutic Chemical
